# Ranibizumab for the treatment of wet AMD: a summary of real-world studies

**DOI:** 10.1038/eye.2016.202

**Published:** 2016-09-09

**Authors:** V Chong

**Correction to:** *Eye* (2016) **30,** 270–286; doi:10.1038/eye.2015.217; published online 4 December 2015

This article was originally published under a standard License to Publish, but has now been made available under a CC BY 4.0 license. The PDF and HTML versions of the paper have been modified accordingly.

